# Changing Therapeutic Paradigms: Predicting mCRC Lesion Response to Selective Internal Radionuclide Therapy (SIRT) based on Critical Absorbed Dose Thresholds: A Case Study

**DOI:** 10.22038/aojnmb.2016.7892

**Published:** 2017

**Authors:** Kathy P Willowson, Elizabeth J Bernard, Richard Maher, Stephen J Clarke, Dale L Bailey

**Affiliations:** 1 Institute of Medical Physics, School of Physics, University of Sydney, Sydney, Australia; 2 Department of Nuclear Medicine, Royal North Shore Hospital, Sydney, Australia; 3 Department of Radiology, Royal North Shore Hospital, Sydney, Australia; 4 Department of Medical Oncology, Royal North Shore Hospital, Sydney, Australia; 5 Faculty of Health Sciences, University of Sydney, Sydney, Australia

**Keywords:** Dosimetry, Liver, Response, SIRT, ^90^Y

## Abstract

A 65 year old male with metastatic colorectal cancer (mCRC) in the liver was referred for selective internal radionuclide therapy (SIRT) following a history of extensive systemic chemotherapy. ^90^Y PET imaging was performed immediately after treatment and used to confirm lesion targeting and measure individual lesion absorbed doses. Lesion dosimetry was highly predictive of eventual response in the follow-up FDG PET performed 8 weeks after therapy. The derived radiation dose map was used to plan a second SIRT procedure aiming to protect healthy liver by keeping absorbed dose below the critical dose threshold, whilst targeting the remaining lesions that had received sub-critical dosing. Again, ^90^Y PET was performed immediately post-treatment and used to derive absorbed dose measures to both lesions and healthy parenchyma. Additional follow-up FDG PET imaging again confirmed the role of the ^90^Y PET dose map as an early predictor of response, and a tool for safe repeat treatment planning.

## Introduction

A 65 year old male with metastatic colorectal cancer (mCRC) in the liver was referred for selective internal radionuclide therapy (SIRT) following a history of extensive systemic chemotherapy. ^90^Y PET imaging was performed immediately after treatment and used to confirm lesion targeting and measure individual lesion absorbed doses. Lesion dosimetry was highly predictive of eventual response in the follow-up FDG PET performed 8 weeks after therapy. The derived radiation dose map was used to plan a second SIRT procedure aiming to protect healthy liver by keeping absorbed dose below the critical dose threshold, whilst targeting the remaining lesions that had received sub-critical dosing. Again, ^90^Y PET was performed immediately post-treatment and used to derive absorbed dose measures to both lesions and healthy parenchyma. Additional follow-up FDG PET imaging again confirmed the role of the ^90^Y PET dose map as an early predictor of response, and a tool for safe repeat treatment planning.

## Case report

We present the case of a 65 year old male with a history of resection for sigmoid colon cancer in December 2008 and systemic chemotherapy (FOLFOX (5FU + oxaliplatin + leucovorin) + Avastin) over the period April – November 2010. Following resection of a solitary liver lesion in April 2011, he recommenced chemotherapy upon recurrence of hepatic disease in June 2012. Upon further progression, he was referred by the Colorectal Cancer Multi-disciplinary Team (MDT) meeting for consideration of SIRT in May 2014. At the time of SIRT work-up there were no serum indicators of hepatic compromise (bilirubin 12 µmol/L (<20 µmol/L), albumin 40 g/L (> 30g/L) and INR normal; WCC 9.9×10^9^/L; Platelets 252×10^9^/L; Creatinine 77 µmol/L) and no inter-current health issues. A liver-lung shunt of 3.3% was estimated from the ^99m^Tc-MAA work-up angiogram, and SPECT imaging showed good localisation of MAA in all lesions identified on the baseline FDG PET. The “modified BSA method” (Sirtex Medical Ltd, Sydney AUS) was used to prescribe 1.56 GBq of ^90^Y for treatment, according to a CT-based segmented tumour burden of 1.1% and a 25% dose reduction due to heavy prior chemotherapy (1).

**Figure 1:**
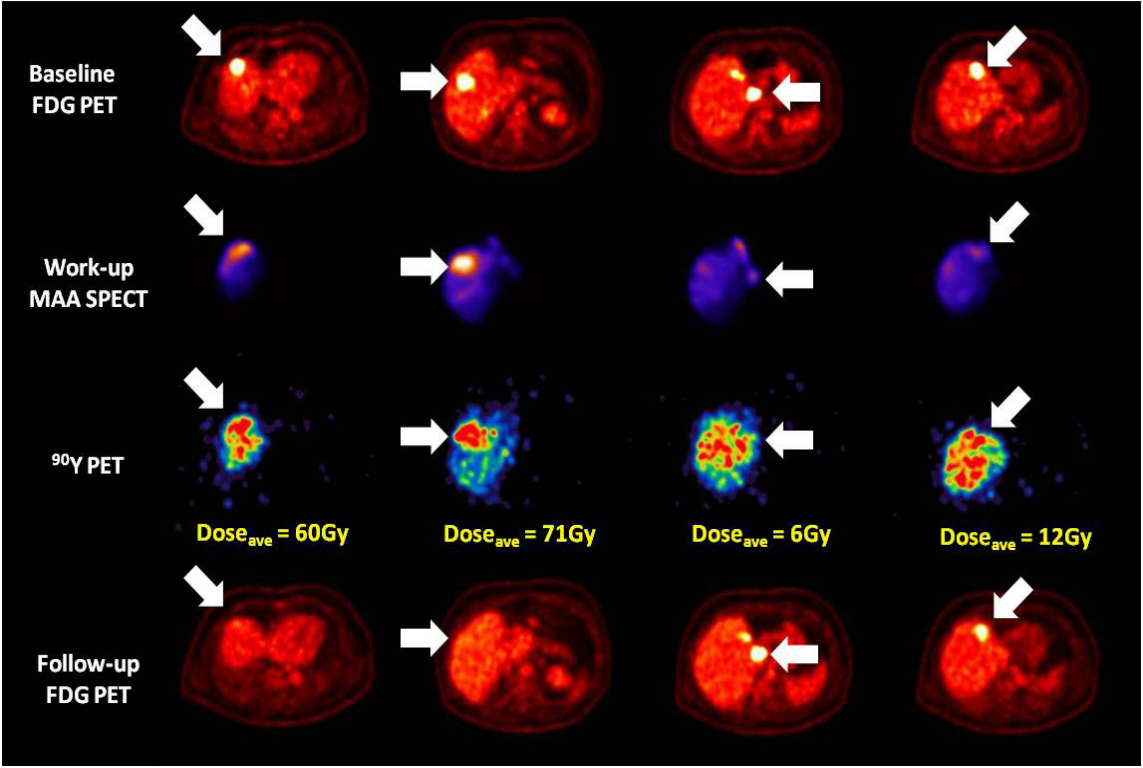
Direct comparison of transverse slices on the baseline FDG PET (upper row), work-up MAA SPECT (second row), ^90^Y PET derived dose map (third row), and 8 weeks follow-up FDG PET (bottom row) for each of the four lesions identified for SIRT. Lesions receiving an absorbed dose above the critical threshold can be seen to exhibit a complete response at the time of follow-up, whereas those receiving sub-threshold dosing exhibit stable disease

**Figure 2:**
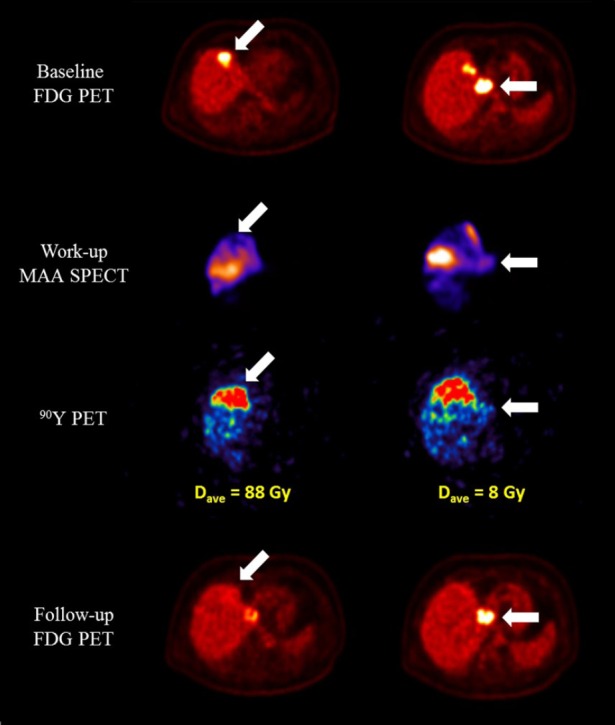
Direct comparison of transverse slices on the baseline FDG PET (upper row), work-up MAA SPECT (second row), ^90^Y PET derived dose map (third row), and 8 weeks follow-up FDG PET (bottom row) for each of the two remaining lesions identified for a second SIRT treatment. The lesion receiving an absorbed dose above the critical threshold can be seen to exhibit a complete response at the time of follow-up, whereas that receiving sub-threshold dosing exhibits stable disease

SIRT was performed 6 days after a baseline FDG PET/CT scan. PET imaging of the in vivo therapeutic distribution of [^90^Y]-SIR-Spheres resin microspheres was acquired 19 hours after implantation, and predicted mean absorbed dose estimates of 6 Gy, 12 Gy, 71 Gy and 60 Gy to the lesions in segments 1, 4a, 5 and 8, respectively. An average of 25 Gy was measured in healthy liver (no area reached 80 Gy, the tolerance level for normal liver quoted in the Sirtex training manual). Follow-up FDG PET was acquired 8 weeks post-SIRT, demonstrating a complete metabolic response for lesions in segment 5 and 8, and stable disease (less than a 50% change in TLG) for lesions in segments 4a and 1 (Figure 1). After further FDG PET imaging in October 2014 indicated progression of disease in the two remaining lesions, a second SIRT treatment was performed in November 2014 (1.56 GBq). Dosimetry again was predictive of response, with lesions receiving 88 Gy and 8 Gy demonstrating a complete response and stable disease, respectively, at the time of follow-up in January 2015 (Figure 2). Healthy liver received an average absorbed dose of 21 Gy (a cumulative dose of 46 Gy over both treatments), with a volume of 10% >80 Gy. No hepatic toxicity or indicators of radioembolisation induced liver disease (REILD) (2) were recorded as of follow-up at 21 months post (second) treatment.

## Discussion

^90^Y SIRT relies on preferential perfusion from the hepatic arteries of target lesions within the liver, based on the premise that healthy liver derives the majority of its blood supply from the portal vein, and so should be spared from large absorbed radiation doses. It has only been in recent years with the advent of ^90^Y PET (3) that the localisation of radiation dose to lesions, and absence in healthy liver or other vulnerable structures (gallbladder, duodenum, etc), can be confirmed post-therapy. The derivation of quantitative dose maps from ^90^Y PET data (4) can act as an early indicator of response to SIRT, if dose-response thresholds are known, and may be an indicator for concurrent alternative therapy targeting those lesions which are under-dosed. In particular, ^90^Y dose maps play an integral part in the safe guiding of further SIRT procedures, where healthy liver parenchyma is at risk of further radiation insult and the development of REILD. A recent report by Bailey et al suggested that mCRC lesions may require a minimum average absorbed dose of at least 30 Gy (5) to reach a significant response, whilst a large prospective study from van den Hooven et al (6) established a dose of 40-60 Gy as the minimum required effective tumour absorbed dose. In terms of the response of normal liver parenchyma to radiation from SIRT procedures, it is speculated that due to the microscopic heterogeneity of the dose in healthy liver (7), a higher absorbed dose may be tolerated than previously thought when compared to external beam radiotherapy uniform dose fields. However, the literature lacks a uniform approach to measures of radionuclide dosimetry and response, and a large scale study would assist in establishing reliable thresholds that may be used to guide treatment planning.
